# Systemic factors associated with 10-year glaucoma progression in South Korean population: a single center study based on electronic medical records

**DOI:** 10.1038/s41598-023-27858-z

**Published:** 2023-01-11

**Authors:** Jung Suk Yoon, Ye-eun Kim, Eun Ji Lee, Hyunjoong Kim, Tae-Woo Kim

**Affiliations:** 1 Department of Ophthalmology, Seoul National University Bundang Hospital, Seoul National University College of Medicine, 82, Gumi-ro, 173 Beon-gil, Bundang-gu, Seongnam, Gyeonggi-do Republic of Korea 13620; 2 Department of Applied Statistics, Yonsei University, Seoul, Republic of Korea

**Keywords:** Diseases, Risk factors

## Abstract

Glaucoma is a multifactorial disease where various systemic features are involved in the progression of the disease. Based on initial systemic profiles in electronic medical records, this study aimed to develop a model predicting factors of long-term rapid retinal nerve fiber layer (RNFL) thinning over 5 years in 505 patients with primary open-angle glaucoma. Eyes with faster or slower RNFL thinning were stratified using a decision tree model, and systemic and ophthalmic data were incorporated into the models based on random forest and permutation methods, with the models interpreted by Shapley additive explanation plots (SHAP). According to the decision tree, a higher lymphocyte ratio (> 34.65%) was the most important systemic variable discriminating faster or slower RNFL thinning. Higher mean corpuscular hemoglobin (> 32.05 pg) and alkaline phosphatase (> 88.0 IU/L) concentrations were distinguishing factors in the eyes with lymphocyte ratios > 34.65% and < 34.65%, respectively. SHAP demonstrated larger baseline RNFL thickness, greater fluctuation of intraocular pressure (IOP), and higher maximum IOP as the strongest ophthalmic factors, while higher lymphocyte ratio and higher platelet count as the strongest systemic factors associated with faster RNFL thinning. Machine learning-based modeling identified several systemic factors as well as previously acknowledged ophthalmic risk factors associated with long-term rapid RNFL thinning.

## Introduction

Glaucoma is a multifactorial disease^1^. Elevated intraocular pressure (IOP) and decreased blood flow are considered the two strongest factors associated with the pathogenesis of glaucoma. However, neither seems to fully explain the mechanism of glaucomatous optic neuropathy, because disease progression is still observed in patients who receive extensive treatment and those lacking evident factors for progression. The identification of factors other than IOP and blood flow may contribute to a greater understanding of the pathogenesis of glaucoma.

Population-based studies have identified several systemic and demographic factors that may be associated with glaucoma. For example, both the Barbados Eye Study^2^ and the Rotterdam study^3^ found that old age, male gender, and family history of glaucoma were associated with the prevalence of open-angle glaucoma (OAG). The Baltimore Eye Survey identified African race, systemic hypertension, low perfusion pressure, and family history of glaucoma as factors associated with the development of glaucomatous damage^4^, whereas the Blue Mountains Eye Study reported that old age, diabetes mellitus^5^, systemic hypertension^6^, and thyroid disease^7^ were associated with the prevalence of OAG. Moreover, the large randomized Collaborative Normal-Tension Glaucoma Study found that female gender, migraine, and African race were risk factors for the progression of normal tension glaucoma (NTG)^8^, and several other studies reported that old age, low blood pressure, and primary vascular dysregulation were risk factors for glaucomatous progression^9–12^. These population-based studies, however, have been limited by their incorporation of relatively few variables associated with risk, with some of these variables based on subjective evaluation by patients. Thus, these studies have been unable to analyze systemic risk factors comprehensively and objectively for glaucoma progression.

Electronic medical records (EMRs) provide large-scale medical databases that are readily available for systematic studies investigating risk factors for various diseases. Our institute has developed an EMR system, beginning in 2009, containing longitudinal data on large numbers of patients. This database is readily available for studies of ophthalmic and systemic factors associated with glaucoma progression. The present study sought to use the large-scale systemic database containing EMRs to identify systemic factors associated with long-term glaucoma progression by machine learning methods.

## Results

The study initially included 756 glaucomatous eyes that satisfied the inclusion criteria. Fifty-seven of the 80 identified variables that showed an importance value < 0.01 in the Random Forest (RF) were excluded by the stepwise method and the remaining 23 variables having an importance value ≥ 0.01 were included in the model. Of the 756 glaucomatous eyes, 251 were excluded because of missing data, resulting in a final sample of 505 eyes. The mean follow-up period was 9.6 ± 1.4 years (range 5.3–11.4 years). The clinical characteristics of the enrolled subjects are summarized in Table 1.

**Table 1:** Clinical characteristics of patients. Values are shown in mean ± standard deviation (range) unless otherwise indicated.

**Demographic characteristics**		
Age (years)	56.1 ± 14.2	(8–82)
Gender (male/female, n)	152 / 138	
**Eye-specific characteristics**		
Axial length (mm)	24.3 ± 1.4	(21.34–28.42)
Central corneal thickness (μm)	549.1 ± 39.8	(385–658)
IOPmean (mmHg)	12.2 ± 2.2	(5.6–22.9)
IOPfluc (mmHg)	2.1 ± 1.4	(0–9.3)
IOPmax (mmHg)	18.8 ± 6.7	(10–52)
Baseline VF MD (dB)	− 6.0 ± 7.1	(− 31.51 to 3.95)
Baseline VF PSD (dB)	5.7 ± 4.4	(1.02–15.57)
Baseline global RNFL thickness (μm)	77.4 ± 17.8	(33–133)
Number of SD-OCT scans	15.5 ± 5.4	(5–36)
Follow-up period (yrs)	9.6 ± 1.4	(5.3–11.4)
Rate of global RNFL thinning (μm/yr)	− 0.83 ± 0.85	(− 4.72 to 0.67)
**Systemic characteristics**		
ALP (IU/L)	73.8 ± 35.3	(24–333)
Cholesterol (mg/dL)	194.1 ± 40.5	(101–438)
Glucose (mg/dL)	110.4 ± 37.9	(72–369)
Lymphocyte (%)	35.1 ± 9.4	(9–67)
MCH (pg)	30.7 ± 1.9	(19.7–34.7)
MCV (fL)	91.8 ± 4.6	(68.6–101.2)
Monocyte (%)	6.8 ± 2.3	(2–17.4)
PCT (%)	0.25 ± 0.07	(0.09–0.7)
PLT (× 10^3^/μL)	241.9 ± 58.6	(80–578)
Urine pH	6.4 ± 0.9	(5–8.5)

*IOP* intraocular pressure, *IOPmean* mean IOP, *IOPfluc* IOP fluctuation, *IOPmax* maximum IOP, *VF* visual field, *MD* mean deviation, *PSD* pattern standard deviation, *RNFL* retinal nerve fiber layer, *SD-OCT* spectral-domain optical coherence tomography, *ALP* alkaline phosphatase, *IU* international unit, *MCH* mean corpuscular hemoglobin, *MCV* mean corpuscular volume, *PCT* procalcitonin, *PLT* platelet.

**Figure 1:**
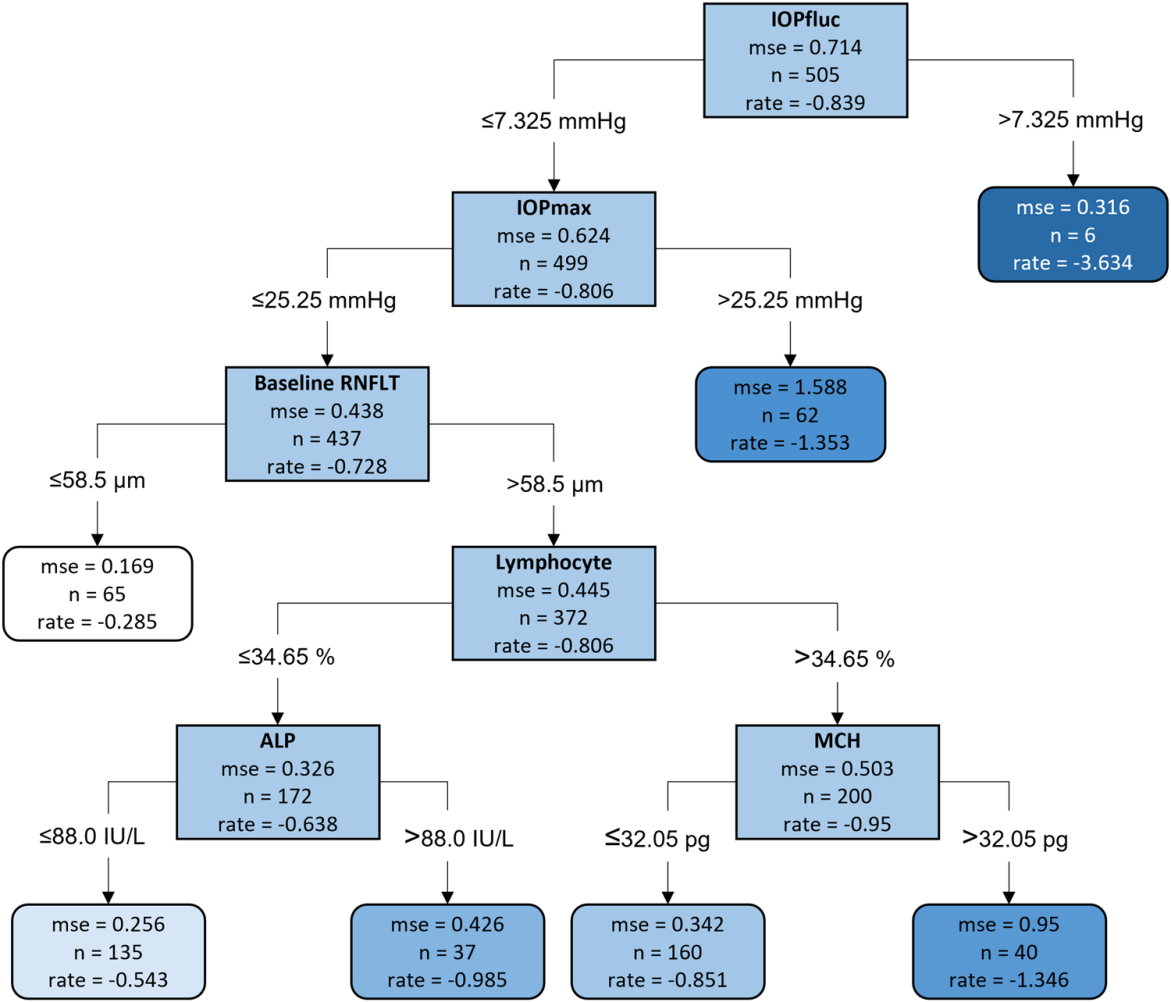
The decision tree model stratifying groups with faster or slower RNFL thinning based on the ophthalmic and systemic variables contributing to the rate of global RNFL thinning. The *rate* in each box indicates the rate of RNFL thinning in microns/year.* IOPfluc* IOP fluctuation during follow-up, *mse* mean squared error, *IOPmax* maximum IOP, *RNFLT* retinal nerve fiber layer thickness, *ALP* alkaline phosphatase, *MCH* mean corpuscular hemoglobin.

A decision tree model was used to stratify patients with faster or slower optical coherence tomography (OCT) retinal nerve fiber layer (RNFL) thinning, which identified six strongly discriminating variables, resulting in seven groups of eyes with different rates of RNFL thinning (Fig. 1). The three strongest variables were ophthalmic variables; eyes with IOP fluctuation (IOPfluc) > 7.325 mmHg (n = 6), maximum IOP (IOPmax) > 25.25 mmHg (n = 62), and baseline retinal nerve fiber layer thickness (RNFLT) > 58.5 μm (n = 65) were more likely to progress rapidly, with RNFL thinning rates of − 3.634 μm/year, − 1.353 μm/year, and − 0.806 μm/year, respectively. The model also identified three systemic discriminating variables for the eyes (n = 437) with smaller IOPfluc, lower IOPmax, and larger baseline RNFLT. The strongest systemic variable was blood lymphocyte ratio; the 200 eyes with blood lymphocyte ratio > 34.65% showed a faster rate of RNFL thinning (− 0.95 μm/year) than the 172 eyes with blood lymphocyte ratio ≤ 34.65% (− 0.638 μm/year). Analysis of the 200 eyes with blood lymphocyte ratio > 34.65% showed that the 40 eyes with mean corpuscular hemoglobin (MCH) > 32.05 pg had a faster rate of RNFL thinning (− 1.346 μm/year) than the 160 eyes with MCH ≤ 32.05 pg (− 0.851 μm/year). On the other hand, the analysis showed that the rate of RNFL thinning in the 172 eyes with a lower lymphocyte ratio (≤ 34.65%) was influenced by serum alkaline phosphatase (ALP), with faster thinning rates observed in the 37 eyes with serum ALP > 88.0 IU/L (− 0.985 μm/year) than in the 135 eyes with serum ALP ≤ 88.0 IU/L (− 0.543 μm/year).

**Figure 2:**
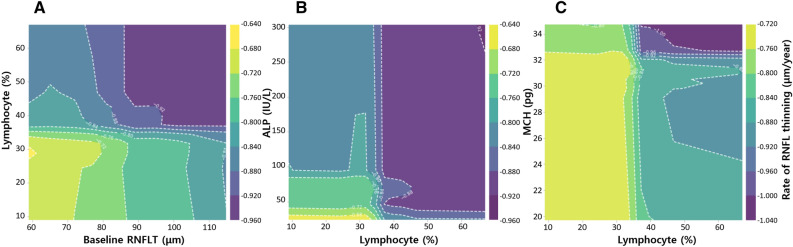
Partial interaction plots showing interactions between two selected factors that affect the rate of RNFL thinning. The scale bar on the right indicates the rate of RNFL thinning. Interactions between baseline RNFLT and lymphocyte ratio (**A**), lymphocyte ratio and ALP (**B**), and lymphocyte ratio and MCH (**C**). These results indicate correlation, not causality. *RNFL* retinal nerve fiber layer, *RNFLT* retinal nerve fiber layer thickness, *ALP* alkaline phosphatase, *MCH* mean corpuscular hemoglobin.

Because systemic factors were significantly associated with RNFL thinning in the fourth node of the decision tree (Fig. 1), the interactions among systemic factors were evaluated in the 372 eyes included in the fourth node (Fig. 1) by partial interaction plots (PIPs) (Fig. 2). The PIPs showed an independent, and strong influence of blood lymphocyte ratio on rapid RNFL thinning when the level exceeded approximately 35%. While the rate of RNFL thinning tended to linearly increase with thicker baseline RNFL (Fig. 2A), the influence of ALP (Fig. 2B) and MCH (Fig. 2C) showed breakpoints (approximately 40 IU/L and 32 pg, respectively), above which the rate of RNFL thinning got faster.

**Figure 3:**
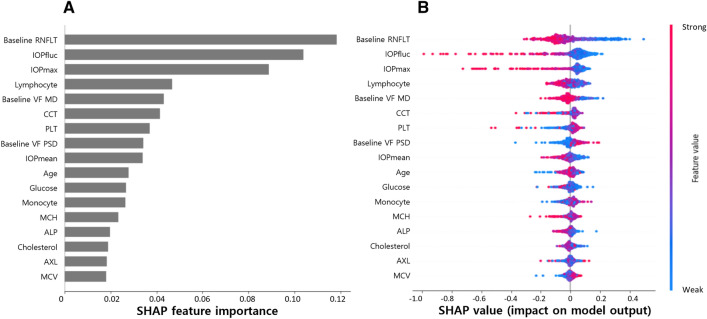
Interpretation of the final model based on ophthalmic and systemic variables. Feature importance plot based on mean SHAP values (**A**). Interpretation of the importance of features using the SHAP plot (**B**). The red and blue colors indicate feature values of high and low levels, respectively. For example, a larger global RNFLT had a strong, negative effect on the rate of RNFL thinning (i.e., faster RNFL thinning). *SHAP* SHapley Additive explanation, *RNFLT* retinal nerve fiber layer thickness, *IOPfluc* intraocular pressure fluctuation during follow-up, *IOPmax* maximum intraocular pressure, *VF* visual field, *MD* mean deviation, *CCT* central corneal thickness, *PLT* platelet, *PSD* pattern standard deviation, *IOPmean* mean intraocular pressure, *MCH* mean corpuscular hemoglobin, *ALP* alkaline phosphatase, *AXL* axial length; *MCV* mean corpuscular volume.

Using RF^13^ and permutation^14,15^ methods, the effects of each variable on the rate of RNFL thinning were assessed, and the results were interpreted using the SHapley Additive exPlanations (SHAP) method. Figure 3 shows a SHAP plot demonstrating the features that strongly predict faster RNFL thinning. The three strongest features were ophthalmic variables, specifically baseline RNFLT, IOPfluc, and IOPmax (Fig. 3A). The SHAP plot showed that larger global RNFLT, larger IOPfluc, and higher IOPmax were associated with faster RNFL thinning (Fig. 3B). Lymphocyte ratio was the strongest systemic variable influencing the rate of RNFL thinning, followed by platelet (PLT) count (Fig. 3A). Higher lymphocyte ratio and lower PLT count were associated with faster rates of RNFL thinning (Fig. 3B).

**Figure 4:**
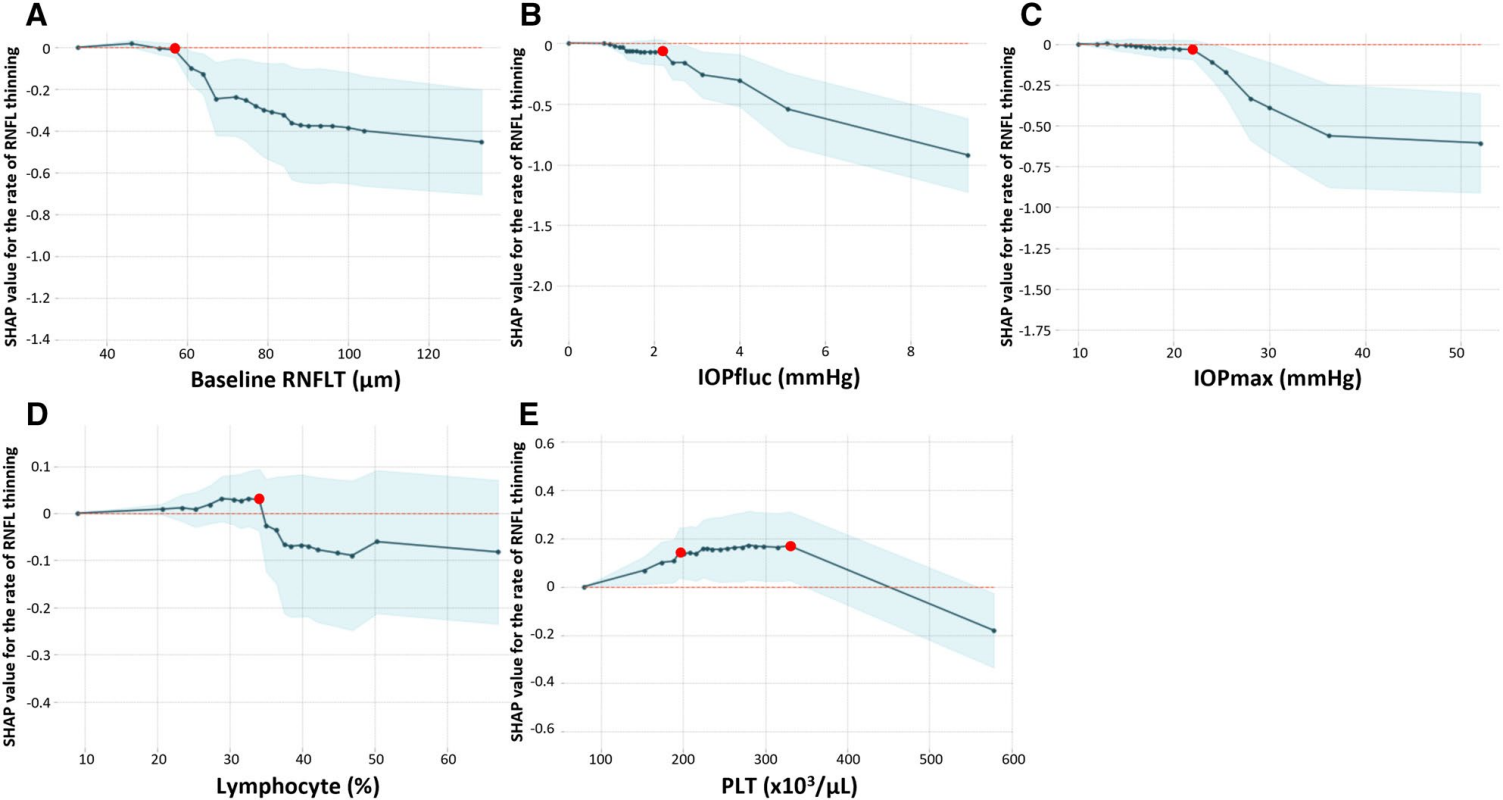
Partial dependence plots of common ophthalmic (**A–C**) and systemic (**D, E**) variables that are found to have the strongest effects on the rate of RNFL thinning by SHAP. Red dots indicate inflection points. *RNFLT* retinal nerve fiber layer thickness, *IOPfluc* intraocular pressure fluctuation during follow-up, *IOPmax* maximum intraocular pressure, *PLT* platelet.

Figure 4 shows the partial dependence plots (PDPs) of the ophthalmic (Fig. 4A–C) and systemic (Fig. 4D, E) variables that had the strongest impact on the rate of RNFL thinning in this model. Inflection points of the PDP curves for baseline RNFLT (Fig. 4A), IOPfluc (Fig. 4B), and IOPmax (Fig. 4C) were 57.0 μm, 2.22%, and 22 mmHg, respectively, indicating that values exceeding these cutoff points were associated with a faster rate RNFL thinning. Lymphocyte ratio > 34.08 (Fig. 4D), and PLT count < 196.0 or > 330.0 (Fig. 4E) were also associated with faster RNFL thinning.

## Discussion

This study was based on a large database containing the EMRs of patients with primary open-angle glaucoma (POAG) who had been followed up for a mean of 10 years. The database contained a wide range of potential systemic predictors of glaucoma progression, with the effects of these factors on the rate of progressive RNFL thinning quantified by various models. In addition to detecting ophthalmic factors, the models revealed several blood test parameters associated with the rate of RNFL thinning. These results suggested that systemic data in patient EMRs can predict glaucoma progression in patients with POAG.

Conventional regression models assume that all relationships are linear and that the interactions between variables are not of interest. These assumptions, however, are not true in real-world applications. The strength of machine learning models is their consideration of all potential nonlinear relationships and interactions among features, enabling the development of more realistic prediction models. The RF and permutation methods are recognized as the most powerful machine learning methods in the development of disease prediction models. The major limitation of these methods has been their high complexity, limiting the ability to interpret their results. However, the recent development of explainable artificial intelligence (i.e., SHAP) has facilitated the interpretation of the results of machine learning models. SHAP allowed the successful explanation of the prediction models, as well as determining the importance and dependence of each feature. The interactions between features could be readily illustrated by partial interaction plots. Another weakness of the machine learning methods is that they do not show the interaction between the features that could together contribute to the result. Despite that the RF is operated by the construction of multiple decision trees in the learning phase, a decision tree model itself can illustrate groups having similar characteristic features that may have interactions while affecting the dependent variable. Using the decision tree, we could stratify patients having different rates of RNFL thinning with different dominant features that may co-contribute to the rate of progressive RNFL thinning.

The decision tree analysis revealed that IOPfluc, IOPmax, and baseline RNFLT were the three variables most predictive of the rate of RNFL thinning. The SHAP feature importance model also showed that these three variables were the most important factors influencing the rate of RNFL thinning. IOP^16,17^ and its fluctuations^18–20^ have been identified as risk factors for glaucoma progression. Although this study focused on systemic factors, the results emphasize the importance of IOP-related variables as predictors of glaucomatous damage. Thicker baseline RNFL has been associated with faster RNFL thinning^21,22^. Because the rate of progression was calculated based on absolute, not percentage, RNFLT, the rate of RNFL thinning should be slower in eyes with thinner baseline RNFL when the same proportion of axons disappears^23^. It is also possible that, due to residual connective tissue^24^, which acts as a floor, RNFLT is unlikely to be below 40 μm, even in eyes with total axonal loss^25^. Therefore, eyes with thin RNFL on OCT progress more slowly than eyes with thicker RNFL.

The decision tree model showed that blood lymphocyte ratio, MCH, and ALP were important systemic variables determining the rate of RNFL thinning. Of these variables, the blood lymphocyte ratio was the strongest, a finding confirmed by SHAP. Inflammatory processes have been reported to be involved in the mechanism of glaucomatous optic neuropathy. For example, evaluations of aqueous humor^26^ and blood samples^27,28^ have found that inflammatory cytokine concentrations are elevated, and T-cell homeostasis altered in patients with POAG. These abnormalities in immune mediators suggest that abnormal immune responses may contribute to the initiation or exacerbation of glaucomatous damage^29^. Abnormal T-cell activity has been associated with glaucomatous degeneration of retinal ganglion cells (RGCs) in rodents^30–32^, and transient elevation of IOP was found to induce T-cell infiltration into the retinas of mice deficient in T and/or B-cells^30^. Taken together, these findings suggest that elevated serum lymphocyte ratio may indicate altered immunity, which may make the optic nerve more susceptible to glaucomatous damage.

Higher MCH was the second systemic determinant associated with faster RNFL thinning in the decision tree model. MCH represents the average quantity of hemoglobin in a single red blood cell. Thus, a decrease in blood MCH may indicate chronic anemic conditions, such as iron deficiency, whereas an increase in MCH may be associated with vitamin B12 (cobalamin) or B9 (folate) deficiency. The relationships between vitamin B12 / B9 deficiency and glaucoma vary among studies. For example, serum concentrations of vitamins B12 and B9 were found to be significantly lower in patients with than without OAG^33^. Other studies, however, found that the serum concentrations of vitamins B12 and B9 did not differ between patients with healthy and glaucomatous eyes^34–36^. The present study found that MCH was an important variable in certain conditions, including those with lower IOPfluc and IOPmax, larger baseline RNFLT, and higher lymphocyte ratio. These findings suggest that discrepancies among study results may be attributable to differences in subject characteristics. Vitamins B12 and B9 are both essential cofactors in homocysteine metabolism, and their insufficiency results in the accumulation of homocysteine, a neurotoxin that can induce RGC apoptosis via stimulation of the N-methyl-D-aspartate (NMDA) receptor^36^. Serum homocysteine concentrations have been reported to be elevated in serum and plasma samples from patients with OAG^35,36^. Moreover, the peripapillary RNFL in the temporal quadrant was thinner in patients with vitamin B12 deficiency than in healthy controls^37^. In contrast, higher MCH was more frequently observed in glaucoma patients with a longer duration than a shorter duration of disease^38^. The present study did not evaluate serum vitamin B12 / B9 or homocysteine concentrations. Thus, the effect of any inter-relationships among MCH, vitamin B, and homocysteine on glaucomatous RGC damage is not conclusive. However, our findings, together with previous results, suggest that high MCH could contribute to progressive RGC damage, under specific conditions.

The decision tree model identified ALP as the second systemic determinant. ALP, an enzyme involved in the hydrolysis of phosphate monoesters, is synthesized in bones, intestines, the placenta, and the hepatobiliary system^39^. Although serum ALP is metabolically inert, elevated serum ALP may indicate disorders involving the hepatobiliary system, bones, or blood. The role of elevated ALP in glaucomatous optic neuropathy is unclear. ALP concentrations were found to be significantly higher in the aqueous humor of eyes with POAG than in nonglaucomatous eyes^40^. Moreover, ALP activity was found to be higher in the trabecular meshwork tissue of glaucomatous eyes^41^. Because ALP is also regarded as a marker of calcification^42,43^, which is involved in the inactivation of mineralization inhibitors^44^, increased ALP activity in the trabecular meshwork might be indicative of a mineralization process during the development of glaucoma^41^. To our knowledge, this study is the first to identify serum ALP as a factor associated with faster RNFL thinning. High serum ALP was found to be important in patients with lower lymphocyte ratio in the present study, whereas high serum ALP concentration accompanied by low lymphocyte ratio has been associated with end-stage renal disease^45,46^ and cancerous conditions^47–50^. Additional studies are required to clarify the effects of ALP on glaucoma progression, and the mechanism by which serum ALP concentration is higher in patients with glaucoma.

The influence of blood lymphocyte ratio, ALP, and MCH seemed to have sharp cutoff points (approximately 35%, 40 IU/L, and 32 pg, respectively), above which the rate of RNFL thinning became remarkably faster (Fig. 2). Notably, that normal ranges are 20–40%, 44–147 IU/L, and 27.5–33.2 pg for blood lymphocyte ratio, ALP, and MCH, which fall near the cutoff points of respective variables. It can be speculated that these systemic variables become pathogenically meaningful when they are abnormally increased above certain levels.

The SHAP model showed that PLT count was the second strongest systemic factor associated with the rate of RNFL thinning, following the lymphocyte ratio. Abnormal PLT activity has been associated with the development and progression of glaucoma. Relative to controls, PLT activity was reported to increase significantly in eyes with POAG^51^, and especially in eyes with NTG^52^. PLT aggregation was higher in POAG patients with visual field progression than patients without in patients suspected of having glaucoma^53^. In contrast, PLT count is decreased in POAG, but PLT distribution width (PDW), a marker indicating a variation in PLT size, was associated with disease severity in patients with POAG^54^. Altered PLT aggregation may have a negative influence on blood flow in small branches of the short ciliary arteries supplying the optic disc^53,55^. In addition, aqueous outflow can be blocked by coagulated PLT in Schlemm’s canal^56^. Our PDP showed that both lower and higher PLT counts were associated with a faster rate of RNFL thinning, whereas values from the 20th to the 95th percentile of the average value were not. It can be speculated that abnormally high and low PLT both play a role in glaucomatous optic neuropathy. However, the influence of PDW on the rate of RNFL thinning was minimal in the present study. Although increased PDW indicated a worse disease stage in the glaucoma continuum^54^, it may not be a causal factor for disease progression. Further study is needed to clarify the causal relationship between PLT activity, PDW level, and glaucomatous progression.

On the other hand, PLT count was found significant only by the SHAP model but not by the decision tree model. A limitation of the decision tree model is that the importance of a variable may be weakened in subgroups after dividing the data by other variables that appear first in the analysis. The decision tree in our study did not find an association between PLT count and the rate of RNFL thinning after separating the eyes into subgroups with a cutoff lymphocyte ratio of 34.65%. RF and permutation methods do not rely on a single decision tree by combining multiple decision trees to create a model.

The strength of this study was that it was based on a large population that had been evaluated over a long period. This study, however, had several limitations, including its retrospective study design and potential selection bias, which may have been caused by the exclusion of some eyes with missing data during processing. In addition, this study was performed to determine the risk factors affecting rapid RNFL thinning within a limited cohort of selected glaucoma patients. Therefore, the results of the study should be validated by further experiments based on a larger database of a prospective study. Another limitation was that all participants were of South Korean ancestry; thus, these results may not be applicable to all ethnic populations. Larger, longer, prospective multicenter longitudinal studies including patients of different ethnic groups are required to confirm these findings.

In conclusion, this study identified some blood test results associated with long-term progressive RNFL thinning in patients with POAG. Identifying a systemic factor prognostic for faster glaucoma progression may enable clinicians to take steps to retard progression in patients predicted to progress despite IOP control. The machine learning approach used in the present study should be considered in any future attempt to discover new risk factors associated with glaucoma and to potentially test whether the systemic factors found in this study could be generalized to other populations.

## Materials and methods

### Participants and ophthalmic evaluation

This study involved the development of machine learning models based on retrospective data contained in patient EMRs of patients diagnosed with POAG between September 17, 2009, and February 11, 2014, at the Glaucoma Clinic of Seoul National University Bundang Hospital. The study protocol was approved by the Institutional Review Board of Seoul National University Bundang Hospital (B-2111-723-104) and adhered to the Declaration of Helsinki. Informed consent was waived by the Institutional Review Board of Seoul National University Bundang Hospital due to the retrospective nature that makes informed consent unfeasible.

At enrollment, all participants underwent comprehensive ophthalmic examinations, including assessments of best-corrected visual acuity (BCVA), Goldmann applanation tonometry, refraction tests, slit-lamp biomicroscopy, gonioscopy, dilated stereoscopic examination of the optic disc, disc photography, red-free fundus photography (EOS D60 digital camera; Canon, Utsunomiyashi, Tochigiken, Japan), spectral-domain optical coherence tomography (SD-OCT) scanning of the circumpapillary RNFL and the optic nerve head (ONH; Spectralis; Heidelberg Engineering, Heidelberg, Germany), and standard automated perimetry (Humphrey Field Analyzer II 750; 24-2 Swedish interactive threshold algorithm, Carl Zeiss Meditec, Dublin, CA, USA). Other ophthalmic examinations included measurement of corneal curvature (KR-1800; Topcon, Tokyo, Japan), central corneal thickness (Orbscan II; Bausch & Lomb Surgical, Rochester, NY, USA), and axial length (IOLMaster version 5; Carl Zeiss Meditec). Mean IOP (IOPmean) was defined as the average of all IOP values measured during the entire follow-up period, except for those measured within the first 6 months after starting IOP lowering medication. IOPfluc and IOPmax were defined as the standard deviation, and the highest IOP of the values that were used to calculate IOPmean, respectively.

Patients were included if they were diagnosed with POAG between September 17, 2009, and February 11, 2014, if their EMRs included systemic test results obtained within 6 months from the time of glaucoma diagnosis, and if they were followed up in the glaucoma clinic for > 5 years and underwent annual SD-OCT examinations to measure circumpapillary RNFL thickness.

A diagnosis of POAG was based on gonioscopy showing an open iridocorneal angle and signs of glaucomatous optic nerve damage (e.g., neuroretinal rim thinning, notching, or a RNFL defect) with consistent visual field defect. Glaucomatous visual field defect was defined as (1) outside normal limits on glaucoma hemifield tests, (2) a cluster of three or more non-edge points on a pattern deviation plot with a probability of < 5%, with one having a probability of < 1%, and/or (3) a < 5% probability of pattern standard deviation confirmed on two consecutive reliable tests, with fixation loss rates ≤ 20% and false-positive and false-negative error rates ≤ 25%.

Eyes were excluded if they had a BCVA worse than 20/40; a spherical equivalent ≤ 8.0 D or ≥ 3.0 D; a cylinder correction ≤ 3.0 D or ≥ 3.0 D; a history of intraocular surgery, except for uneventful cataract surgery; or a retinal (e.g., diabetic retinopathy, retinal vessel occlusion, or retinoschisis) or neurological (e.g., pituitary tumor) disease. When both eyes were eligible, one eye was randomly selected for this study.

### EMR data source

Systemic data obtained within 1 year from the first OCT examination was extracted from the EMRs. These systemic evaluations included the results of regular health screenings, tests from other departments, or preoperative checkups. Factors evaluated included complete blood counts, such as absolute neutrophil counts (/μL), the percentages of atypical lymphocytes (%), band neutrophils (%), basophils (%), eosinophils (%), large unstained cells (%), lymphocytes (%), metamyelocytes (%), monocytes (%), myelocytes (%), normoblasts (%), and segmented neutrophils; erythrocyte sedimentation rate (mm/hr), hemoglobin concentration (g/dL), hematocrit (%), mean corpuscular hemoglobin (MCH, pg), mean corpuscular hemoglobin concentration (g/dL), mean corpuscular volume (MCV, fL), mean plasma volume (fL), procalcitonin (PCT, %), platelet distribution width (PDW, %), platelet (PLT, × 10^3^/μL), red blood cell (× 10^6^/μL), white blood cell (× 10^3^/μL) counts, and red cell distribution width (as both coefficient of variation (%) and standard deviation (fL). Serum chemistry tests included serum concentrations of albumin (g/dL), alkaline phosphatase (ALP, IU/L), aspartate aminotransferase (IU/L), alanine aminotransferase (IU/L), blood urea nitrogen (mg/dL), total bilirubin (mg/dL), total CO_2_ (mmol/L), C-reactive protein (mg/dL), calcium (mg/dL), chloride (mmol/L), cholesterol (mg/dL), creatinine (mg/dL), glucose (mg/dL), phosphorus (mg/dL), potassium (mmol/L), total protein (g/dL), sodium (mmol/L), and estimated glomerular filtration rate. Serologic tests included concentrations of anti-HBs (IU/L), anti-HCV (positive/negative), HBsAg (positive/negative), HIV Ag/Ab (positive/negative), and rapid plasma reagin (reactive/nonreactive). Urinalysis tests included blood (+/−), bilirubin (+/−), color, ketone (+/−), nitrite (+/−), pH, protein (+/−), specific gravity, turbidity, urobilinogen (+/−), and white blood cell stick (+/−). Coagulation tests included prolongation time (%), prolongation time (international normalized ratio), prolongation time (seconds), and activated partial thromboplastin time (seconds).

To exclude variables with least importance, those with an importance value < 0.01 in the RF model were deleted by repeated calculations.

### Determination of the rate of OCT RNFL thinning

Circumpapillary RNFLT was measured using a circular scan protocol of the Spectralis OCT system (Heidelberg Engineering, Heidelberg, Germany). Measurements at baseline were obtained by placing a circle of diameter 3.5 mm concentric with the optic disc. Follow-up scans were obtained using a built-in realignment procedure. The rate of change of global RNFLT (expressed as microns per year) for each subject was determined by linear regression analysis over time.

Only images with adequate quality of Spectralis Q (signal strength) > 15 were included. Images with motion artifacts, missing data, and/or centration errors were excluded. The accuracy of the segmentation of the RNFL was reviewed, and segmentation errors were manually corrected.

### Data processing

All records in the data source were thoroughly organized and indexed. Raw data were exported to python for processing and analysis. The decision tree model was used to stratify patients with faster or slower RNFL thinning, based on factors influencing the rate of RNFL thinning. The effect of each variable on the rate of RNFL thinning was assessed using random forest (RF)^13^ and permutation^14,15^ methods. Because machine learning models are difficult to interpret due to their complexity, the results of the models were interpreted using the SHAP method^57^, a type of explainable artificial intelligence method. The source code for our machine learning models is available at the GitHub repository (https://github.com/hyunjoongkim1/Systemic-factors-for-long-term-glaucoma-progression/new/main).

### Decision tree analysis

Decision tree models classify variables into two groups by predicting the optimal cut-off value to divide subgroups. These models search for the best predictor and the corresponding cutoff value that splits one group into two subgroups, such that the responses of the two subgroups differ significantly. The present study used a decision tree model to stratify the variables in order of their contribution to the rate of RNFL thinning. All possibilities of nodal splits were considered, with stepwise elimination used to determine the model that best delineated the risk factors associated with a faster rate of RNFL thinning. Other methods, including RF, permutation, and SHAP feature importance, were used to reinforce the results derived from the decision tree and to check for overfitting. Interactions between features were visualized by partial interaction plots.

### Random forest feature importance

RF is an ensemble learning method for classification and regression. During the learning phase, RF constructs multiple decision trees, reducing the risk of overfitting, a weakness of regression trees, by averaging over multiple decision trees^13^. When generating decision trees in RF, various decision trees are created using a random variable selection technique. It is well known that RF produces more accurate predictions than a single decision tree. Although RF has been shown to outperform the predictive performance of other machine learning methods, it is difficult to interpret the results predicted by the RF method, a disadvantage similar to that of other machine learning methods. The number of decision trees for RF learning was set at 200, with five-fold cross-validation showing that the optimal number of features for each node was three.

### Permutation feature importance

The permutation method is a method of exploring the data space that a variable can make by randomly permuting values. The importance of a variable can be evaluated according to how much the predicted value after permutation differs from the value before permutation. If it is a significant variable, the prediction accuracy will be greatly reduced. Permutation feature importance is defined as a decrease in a model score or an increase in prediction error when a single feature value is randomly shuffled. Because this procedure reduces the relationship between the feature and the true outcome, a reduction in the model score is indicative of the magnitude of dependence of the model on the feature. The difference in performance is regarded as the importance of the feature^14,15^.

### SHAP feature importance

The SHAP method was developed to explain the output of any machine learning model. Based on the output of a particular model, SHAP values can represent a fair or reasonable allocation of feature importance. After constructing a model with several features, the SHAP value was obtained by determining the average change relative to the presence or absence of any individual feature. The SHAP value of each feature was an indicator of the strength of that feature on the positive or negative prediction of the model, with a larger absolute SHAP indicating that the feature had a greater impact on prediction by the model^57,58^. SHAP values were calculated to determine the contribution of each variable and its correlation with the rate of RNFL thinning. In addition to the feature importance plot, the SHAP values were used to draw partial dependence plots, evaluating the marginal effects of strong systemic variables on the rate of RNFL thinning^59^.

### Data analysis

Except where stated otherwise, data are presented as mean ± standard deviation. All statistical and machine learning analyses were performed using Python v3.8.5, with the scikit-learn package v1.0 used for regression analysis and decision tree construction and the shap package v0.39.0 used for SHAP analysis.

## Data Availability

The datasets generated during and/or analyzed during the current study are available from the corresponding author upon reasonable request.
